# Physical Behavior in Older Persons during Daily Life: Insights from Instrumented Shoes

**DOI:** 10.3390/s16081225

**Published:** 2016-08-03

**Authors:** Christopher Moufawad el Achkar, Constanze Lenoble-Hoskovec, Anisoara Paraschiv-Ionescu, Kristof Major, Christophe Büla, Kamiar Aminian

**Affiliations:** 1 Laboratory of Movement Analysis and Measurement, Ecole Polytechnique Fédérale de Lausanne (EPFL), 1015 Lausanne, Switzerland; christopher.moufawadelachkar@epfl.ch (C.M.e.A.); anisoara.ionescu@epfl.ch (A.P.-I.); 2 Centre Hospitalier Universitaire Vaudois (CHUV), Service de gériatrie et réadaptation gériatrique, 1011 Lausanne, Switzerland; constanze.hoskovec@chuv.ch (C.L.-H.); kristof.major@chuv.ch (K.M.); christophe.bula@chuv.ch (C.B.)

**Keywords:** activity classification, gait analysis, inertial measurement unit, pressure insole, wearable sensors, behavioral complexity

## Abstract

Activity level and gait parameters during daily life are important indicators for clinicians because they can provide critical insights into modifications of mobility and function over time. Wearable activity monitoring has been gaining momentum in daily life health assessment. Consequently, this study seeks to validate an algorithm for the classification of daily life activities and to provide a detailed gait analysis in older adults. A system consisting of an inertial sensor combined with a pressure sensing insole has been developed. Using an algorithm that we previously validated during a semi structured protocol, activities in 10 healthy elderly participants were recorded and compared to a wearable reference system over a 4 h recording period at home. Detailed gait parameters were calculated from inertial sensors. Dynamics of physical behavior were characterized using barcodes that express the measure of behavioral complexity. Activity classification based on the algorithm led to a 93% accuracy in classifying basic activities of daily life, i.e., sitting, standing, and walking. Gait analysis emphasizes the importance of metrics such as foot clearance in daily life assessment. Results also underline that measures of physical behavior and gait performance are complementary, especially since gait parameters were not correlated to complexity. Participants gave positive feedback regarding the use of the instrumented shoes. These results extend previous observations in showing the concurrent validity of the instrumented shoes compared to a body-worn reference system for daily-life physical behavior monitoring in older adults.

## 1. Introduction

Physical activity and behavior are critical to maintain a healthy long-term lifestyle. Several chronic health conditions and diseases are caused or aggravated by physical inactivity [1], and sedentary behavior (time spent in sitting or lying posture) is linked to higher mortality rates even in relatively active persons [2]. In older adults, increased activity levels can sustain independence and delay the onset of decline [3], and lower fall risk [4].

Today’s standard in activity assessment is shifting from questionnaires to sensor-based technologies, triggered by the poor recall and subjectivity of the former compared to objective measures obtained from the latter [5]. Body worn motion sensors, mainly based on inertial measurement units (IMU) [6,7] offer a pervasive (indoor/outdoor) monitoring. The main challenge remains in the validation of activity classification algorithms relying on wearable sensor data, which are mostly based on machine learning rules, i.e., learning from a training set and extending classification to a testing dataset. Validation procedure is generally performed in laboratory conditions, where performed activities are scripted and annotated by an observer following the participant. Alternatively, validation can be performed freely in daily life without restrictions and without the presence of an observer [8]. Semi-structured data collection protocols were recently recommended whereby the participant performs a series of activities in a lifelike scenario (e.g., walking along a track with stop points for sitting) for at least 30 min at their comfortable speed and in the manner they prefer [9]. This latter type of data collection could be extremely useful for algorithm development before validation in real-life conditions.

The difficulty in validating algorithms to classify activity when using wearable sensors lies in acquiring the ground-truth, i.e., the real activity to be used as reference. To date, three main ground-truth reference systems have been used: video observation [10,11,12,13], direct observation with annotation [14,15], and self-annotation by the participant [16,17,18]. Video and direct observations both enable accurate reporting of activity reference but have several drawbacks. In direct observation, the study investigator has to write down the activities in real time as they occur, and subsequently perform manual labeling by evaluating the sensor signals. This task is highly time-, effort-, and resource-consuming [19]. Moreover, it interferes obtrusively with the regular activities of monitored subjects in their home environment. Video observation also requires tedious post-analysis to label the activities from the recordings and poses inevitable privacy concerns [20]. Additionally, it is recommended that at least two investigators label activity reference from video or direct observation to minimize observer errors [9]. Self-reporting is certainly less intrusive than the two other approaches. However, it can lack accurate activity labeling due to subject forgetfulness and has been shown to misestimate activities as well as, in some cases, to result in over-reporting higher intensity instances [21]. Since most activity monitoring targets populations that are somewhat diseased or at-risk, self-reporting can be unreliable, especially when considering cognitively impaired older persons.

The use of an already validated wearable monitoring system is an alternative to the aforementioned validation techniques that has been applied in other works [22,23]. Validations relying on such systems eliminate the need for an external observer or intrusive video recording, and profoundly reduce post-processing complexity. The ground-truth activity labels can be simply obtained by applying the validated algorithms on collected data. However, participants might be required to wear or carry additional sensors during the validation phase. It is recommended that a wearable reference system have at least 90% sensitivity and specificity for activity classification [9], which has already been demonstrated by some multi-sensor systems [24,25].

Walking is an important activity in daily life. Nevertheless, its assessment is usually performed in the laboratory, using stationary gait analysis systems. Lab-based gait analysis has shown efficacy in fall risk evaluation [26], and fear-of-falling related gait modifications [27]. Gait parameters such as stride velocity and cadence have been associated with mortality [28,29], whereas foot clearance might reveal different obstacle avoidance strategies in young and elderly subjects [30]. Building on lab-based assessment, gait monitoring during daily life has provided promising preliminary results in recent years, including fall prediction and risk estimation [4] as well as insights on the association between fall incidence and gait performance [31]. Nevertheless, due to the predominant sensor configuration (i.e., trunk-attached sensor) in studies of gait under real-life conditions, only a limited number of gait parameters have been studied so far.

The complexity of physical behavior in daily life has been recently revealed by multi-sensor systems combining the different activity determinants, (i.e., FITT principle for frequency, intensity, time, and type), in a barcode and calculating the entropy of the activity barcode [32]. This combination provides a global index of physical behavior and its dynamics. Applying complexity measures in physical behavior analyses has proved very useful in providing improved assessment in patients suffering from chronic pain. The information from activity barcodes is extremely rich and its application to other population, such as elderly persons, could provide complementary information beyond those obtained from classical analyses of physical behavior and gait performance.

Consequently, there is an evident need for an instrument that can combine capturing reliably, easily, and for a long period both the coarse-grained daily activity of older adults in terms of activity type, and the fine-grained gait analysis of locomotion periods. We previously developed instrumented shoes and validated an activity classification algorithm using a wearable reference system and applying a semi-structured activity protocol in healthy elderly subjects [33]. The instrumented shoes system has multiple sensor modalities capable of measuring the load under each foot and its movement, all contained in a single location. A global accuracy of 97% was achieved by using an event-driven algorithm inspired from movement biomechanics, revealing the advantage of using the foot (or shoe) as a single sensor location. In fact, compared to systems with sensors placed on multiple body locations, the algorithm revealed similar activity classification performances. However, the system has so far not been validated in real-life conditions. Furthermore, by recognizing daily walking activity, gait parameters could be estimated using an IMU-based algorithm [34]. Activity barcodes could be built from the activity output of the classification algorithm combined with pertinent gait parameters. Therefore the objectives of this study were, first, to demonstrate the concurrent validity of the instrumented shoes system in classifying basic activity types in real-life conditions. Secondly, we aimed to provide a refined analysis of locomotion periods by presenting clinically relevant gait parameters that until now cannot be obtained routinely outside of a laboratory setting. Finally, the potential of calculating a physical behavior complexity metric using the instrumented shoes was evaluated.

## 2. Materials and Methods

### 2.1. Activity Classification

#### 2.1.1. Instrumented Shoe and Reference Systems

**Figure 1:**
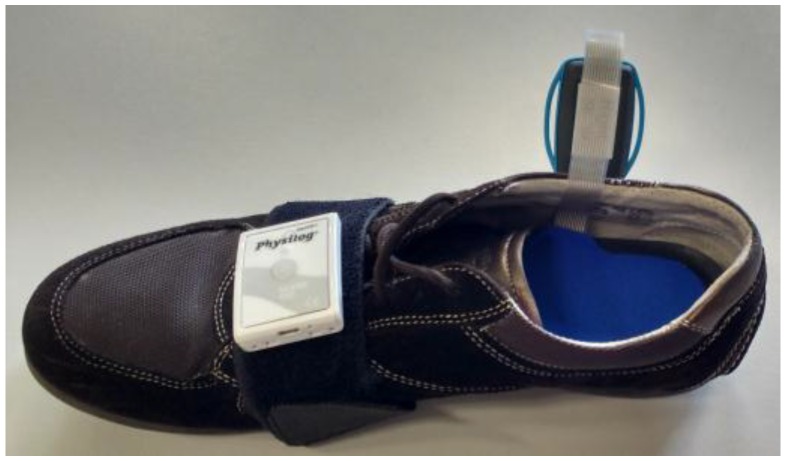
Instrumented shoe system (right shoe). The Physilog^®^ is placed on a strap looping around the shoe with Velcro^®^ tape. The insole (in blue) is placed inside the shoe and linked to the Physilog^®^ by a cable. Converting electronics are in the box with handles (lateral side of the shoe), connected to the strip stemming from the insole.

The instrumented shoe system consists of two main components: a Physilog^®^ inertial measurement unit (IMU) (GaitUp, Lausanne, Switzerland) with 3D accelerometer, 3D gyroscope, 3D magnetometer, temperature and barometric sensor and a force sensing insole (IEE, Luxembourg) that measures the pressure under 8 regions of the foot: hallux, the remaining toes, the first, third and fifth metatarsals’ heads, the lateral longitudinal arch, the lateral and medial heel. The pressure sensing insole is sandwiched between two layers of neoprene for protection, humidity resistance and increased comfort. The complete insole has a thickness of 3 mm. The Physilog^®^ has a thickness inferior to 1 cm and weighs less than 20 g. The system components are shown in Figure 1. All sensors are powered by a battery and data are acquired on a memory card, both integrated in the Physilog^®^ module. The insole data is digitized and amplified by custom-made electronics placed in a separate box. One Physilog^®^ was placed on the dorsal aspect of each shoe and one insole was inserted into each shoe. The box containing the electronics was strapped to the ankle.

Participants were additionally equipped with a reference system consisting of one Physilog^®^ sensor on the right thigh and another on the trunk, both fixed with hypoallergenic tape to minimize discomfort and protect the sensors from humidity. These two sensors were used to provide the reference activity for validation purposes [25]. This reference system has proven high sensitivity and specificity (>90%) in classifying *sitting*, *standing* and *walking*, and has already been used for similar validation purposes in other studies [23,35]. Instrumented shoes and reference systems were synchronized electronically by radio frequency and all data were sampled at 200 Hz, offering an autonomy of more than 16 h.

#### 2.1.2. Participants and Data Collection

Ten healthy community-dwelling elderly participants were recruited for this study, eight men and two women. Overall physical characteristics of this convenience sample were (mean ± standard deviation): age 69.9 ± 3.1 years old, weight 80.1 ± 14.7 Kg, height 171.7 ± 8.9 cm, shoe size range 39–45 EU.

Participants came to the laboratory and were equipped with the instrumented shoes and reference system. Two tests were performed for the purpose of calibration: (a) standing still for 5 s; (b) level walking for 10 straight steps. A semi-structured activity protocol was then followed by each participant, the results of which have already been reported [33]. During this protocol, participants followed a predefined track and performed basic activities (i.e., sitting, standing, and walking) as well as more detailed locomotion types including stair, ramp, and elevator ascent and descent. Participants then returned to their daily activities outside the laboratory after the sensor setup. They were simply requested to keep their shoes on over a 4 h monitoring period, used for the analyses in this study. Once the measurement time had elapsed, a study investigator retrieved the sensors from the participant. No observer followed the participants around, so they were free to perform their activities independently. All data were stored anonymously on a PC for post-processing and analysis. All participants gave written consent to participate and the study was approved by the university’s ethical committee under the title: “Quantification of postural transitions using multimodal sensory input” and reference “EK 2012-N-32”.

#### 2.1.3. Sensors Calibration

Inertial sensors were calibrated in static position to correct for any gain and offset errors by using Ferraris’ method [36]. The sensors were then aligned to the foot frame during a level walking period of 10 steps at the laboratory. The gravity alignment was done during foot static periods (stance phase) and the medio-lateral axis was found as the principal component during swing phase of the foot by assuming that the movement was mainly in the sagittal plane.

Raw pressure data from the insole were calibrated to the body weight (BW). The sum of all 16 sensors from both feet was divided by BW which was obtained during 5 s of static standing initially performed in the laboratory. This provided an estimation of the total force (*TF*) under the feet, Equation (1):

(1) $$TF=\frac{\sum right_{insole\left(i\right)}+\sum left_{insole\left(i\right)}}{BW}$$

where *i* ranges from 1 to 8.

#### 2.1.4. Event-Driven Activity Classification Algorithm

The algorithm is based on a previous study that evaluated the activity classification in a semi-structured protocol [33]. The algorithm is capable of classifying the basic activities such as *sitting*, *standing*, *walking*; and activity subclasses including *stair climbing*, *incline walking*, and *elevator use*. An event-driven classification tree was applied to classify the activities at each node by using data input from the different sensors in the instrumented shoes. The IMU data (accelerometer, gyroscope and barometric pressure sensor) were used to detect *walking* and classify different locomotion types whereas the insole data were employed to distinguish *sitting* from *standing*. *Locomotion periods* were identified by step detection using Toe Off (TO) instants. The pitch angular velocity (foot rotation around the medio-lateral axis) was subjected to a wavelet transform enhancing the TO, as well as other gait events, i.e., mid swing and Heel Strike (HS) instants. A Coiflet order 5 wavelet was used to decompose the signal into 10 scales, and two combinations were used. Subtracting the 9th approximation from the first emphasized HS, while subtracting it from the third emphasized TO [37]. *Stair climbing* and *elevator use* were detected by using barometric pressure, whereas foot inclination from IMU during stance was used for *incline* and *level walking* identification. A threshold on the *TF* estimate was applied on the non-locomotion data to classify *sitting* and *standing*. *Lying* and *sitting* were considered as a single activity type in this study.

#### 2.1.5. Evaluation of the Activity Classification Algorithm

The reference activity classification algorithm combines information from trunk and thigh IMU in order to classify basic activity [23]. In the current study, the validation is mainly intended for these basic activities (*walking*, *sitting/lying*, and *standing*) since there was no reference data for the remaining subclasses. The activity outputs from the instrumented shoes classifier and reference algorithm were segmented into 6 s windows to remove spurious activities. The median activity from the instrumented shoes’ and the reference system’s classification algorithms were compared for each 6 s window and the true positives (TP), true negatives (TN), false positives (FP), false negatives (FN) were obtained. Sensitivity, specificity, precision, F1-score (F-measure) and global accuracy were calculated for each activity class according to the following equations:

$$Sensitivity=\frac{\#TP}{\#TP+\#FN}$$

$$Specificity=\frac{\#TN}{\#TN+\#FP}$$

$$Precision=\frac{\#TP}{\#TP+\#FP}$$

$$F1-score=\frac{2\times precision\times sensitivity}{precision+sensitivity}$$

$$Global\;Accuracy=\frac{\#TP+\#TN}{total\;sample\;number}$$

### 2.2. Gait Analysis

**Figure 2:**
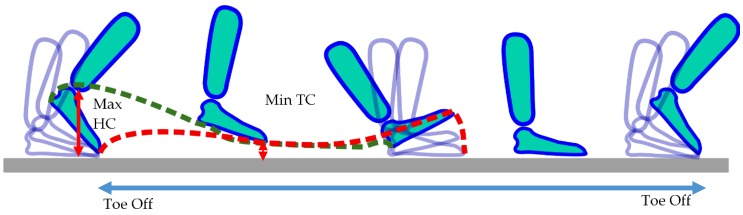
Foot clearance during a step from a single foot. The maximum heel (HC) and minimum toe (TC) clearance are shown with arrows. Two consecutive toe off instants are shown, forming a complete gait cycle.

Locomotion periods obtained through the activity classifier were retained for this specific analysis. The cumulative distribution of locomotion bouts was extracted by taking into consideration any period with 3 or more detected steps, corresponding to a minimum of one gait cycle (e.g., left-right-left or right-left-right step sequences). The minimum of three steps has been applied for gait detection in several other studies [31,38] since this ultimately prevents the algorithm from classifying spurious foot movement. A gait cycle based on the locomotion detection algorithm is defined between two successive TO instants of each foot (Figure 2). Cadence distribution is estimated with a histogram of 1 step/min bins. The number of bouts, total duration and total number of steps are tabulated for upstairs, downstairs, uphill and downhill periods, respectively.

Gait analysis was performed in terms of spatio-temporal parameters i.e., stride velocity, stride length, cadence, inter-stride gait cycle time variability, and foot clearance parameters, i.e., maximal heel clearance (HC), and minimum toe clearance (TC) [39]. HC corresponds to the maximum heel height above the ground at the beginning of the swing phase whereas TC corresponds to the minimum toe height above the ground in the middle of the swing phase [39]. These gait parameters were extracted from locomotion periods with at least 20 steps (combined right and left feet) to achieve steady-state gait [40]. Initiation and turning steps, i.e., steps with a turning angle higher than 20 degrees, were detected [34] but omitted during the parameter extraction since they do not pertain to steady-state gait analysis. Stair and slope locomotion (ground inclination of more than 5% or 3 degrees) was also excluded from the analysis.

### 2.3. Complexity and Activity Barcodes

Activity levels were obtained from the states defined by Paraschiv-Ionescu et al. [32]. In summary, these states start by low levels pertaining to low intensity during *sitting* and *standing*, going to higher levels of activity obtained by combining gait cadence and duration of locomotion periods. Overall, this classification yields 18 ranked states, where each state is represented by a color code, with warmer colors indicating higher activity intensity. The barcodes are based on 1 s-windows represented by a color corresponding to the median of the activity state over the samples forming the window. Previous work has shown that such a barcode has higher color (state) entropy in healthy subjects compared to subjects with pain or disease [32]. Using the outcome of instrumented shoes, activity barcodes were similarly evaluated by using 14 states (represented by numeric codes) instead of 18 (Table 1). This reduction resulted from assigning only a single numeric code to both *sitting* (1) and *standing* (2) whereas, in the original activity barcode, *sitting* and *standing* were assigned 2 and 4 numeric codes, respectively, based on trunk movement intensity. These states were reduced to 2 in the present study to avoid using trunk sensor data and keep the activity barcode specific to the instrumented shoes. *Walking* was segmented into locomotion periods of duration d < 30 s, 30 s < d < 120 s and 120 s < d. For each locomotion period, the mean cadence was calculated in steps/min. The cadence was then segmented into cad < 50, 50 < cad < 80, 80 < cad < 140 and 140 < cad. The combinations of duration and cadence represent 12 numeric codes as shown in Table 1.

The entropy (complexity) of obtained barcodes was estimated using the Lempel-Ziv complexity metric [41,42]. The correlation between the instrumented shoes and reference system complexities was calculated. The correlation between the Lempel-Ziv complexity evaluated from the instrumented shoes and gait parameters such as the stride velocity, stride length, max HC and min TC, as well as the duration of steady-state gait cycles was calculated.

### 2.4. System Comfort Evaluation

Gathering feedback from the system users is important. Therefore, at the end of each data collection, the participants were asked the following question: “On a scale ranging between 0 “not comfortable at all” and 10 “very comfortable”, what score would you give to the system in terms of comfort during daily use?” Scores were recorded by the investigator retrieving the sensors at the end of the monitoring period.

## 3. Results

### 3.1. Activity Classification

**Figure 3:**
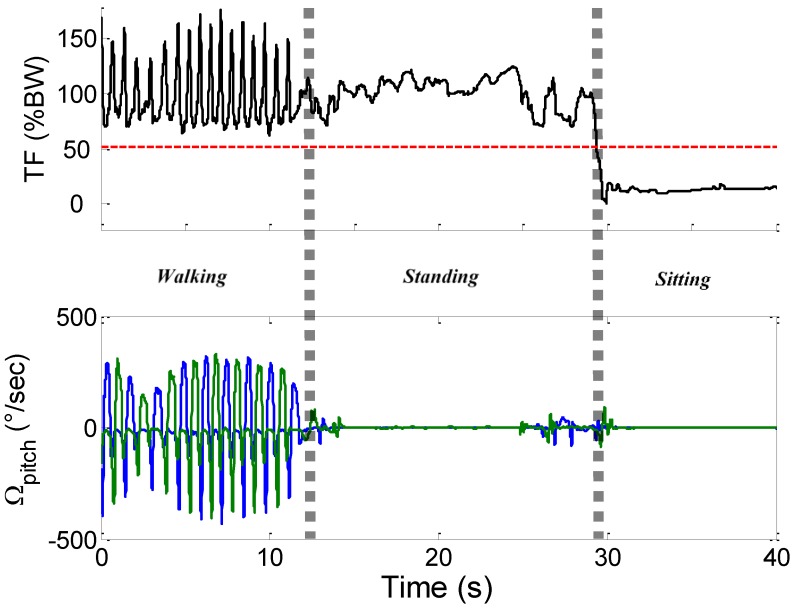
Snapshot of classifier output from one participant (taken ~1 h after the beginning of the recording). Top: plot of TF showing the 50% BW line (dashed red line). Bottom: pitch angular velocity: right foot (blue) and left foot (green). The vertical dashed bars represent different activity periods (walking, standing and sitting).

**Figure 4:**
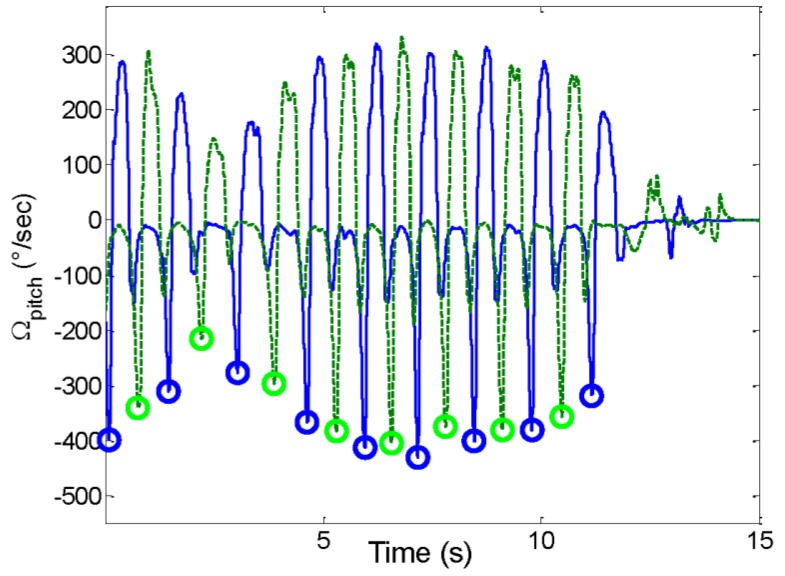
Zoom-in on the walking period from Figure 3. The pitch angular velocity of the right foot is shown as a continuous line, and the left foot as a dashed line. TO instants are represented by circles.

A sample output of the event-based activity classification algorithm is shown in Figure 3. The data are selected from one subject and show a sequence of *walking*, *standing* and *sitting*. The 50% BW line is marked on the figure to show the distinction between *sitting* and *standing*. The TO instants used to classify *walking* are displayed in Figure 4, which is a zoom-in of the same *walking* period from Figure 3.

Table 2 shows the confusion matrix and the classifier performances compared to reference activity. Sensitivity, specificity, precision and F-score were all 90% or higher for all activities except the sensitivity of *standing* (88%). Only 11 *sitting/lying* instances were predicted as *walking*, and one instance of *walking* were predicted as *sitting/lying*. Highest sensitivity was obtained for *sitting/lying* (99%) and highest specificity for *walking* and *sitting/lying* (98% and 99%). A precision of 95% was achieved for *sitting/lying* as well as an F-score of 97%. The algorithm achieved a global accuracy of 93%.

### 3.2. Gait Analysis of Locomotion Periods

**Figure 5:**
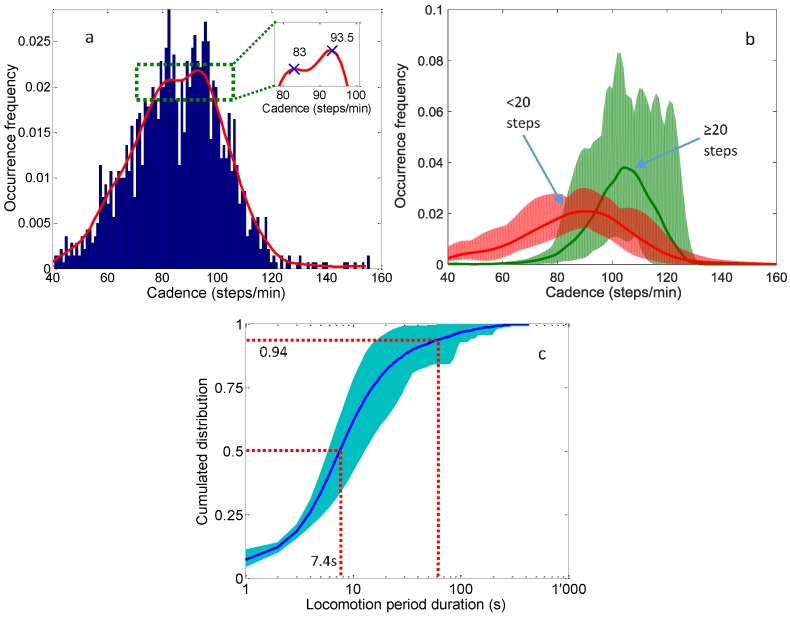
(**a**) Mean cadence distribution for all locomotion periods with three or more steps; (**b**) Instantaneous cadence distribution for locomotion periods with 20 or more steps vs less than 20 steps; (**c**) Cumulative distribution of locomotion period duration across all subjects (log scale for locomotion period duration axis). For (**b**,**c**): mean is represented by a thick line and SD by a shaded area.

Mean cadence for every locomotion period with three or more steps is plotted as a histogram with a bin size of one step/min. A kernel smoothing fit is applied on this histogram as shown in Figure 5a. The two peaks of this fit correspond to a bimodal distribution with mode values of 83 and 93.5 steps/min. The separation of cadence distributions between locomotion periods of 20 or more steps and locomotion periods of less than 20 steps is also shown in Figure 5b to better illustrate the hypothesis that cadence mode during short locomotion bouts is lower. This is done by obtaining the probability density function of each instantaneous cadence distribution per subject and calculating a mean ± SD distribution. The distribution modes in this case are 90 steps/min (less than 20 steps) and 104 steps/min (20 steps or more). These values are somewhat different from the modes obtained for the entire distribution above because of the discrete separation of locomotion periods. The cumulative distribution of locomotion period durations (level and non-level) is shown on a semi-log plot, Figure 5c. The mean (thick line) and SD (shading) describe the locomotion period durations across all subjects. The longest continuous locomotion period was 432 s or 7.2 min. About 50% of locomotion periods lasted less than 7.4 s, and 94% were less than one minute.

Table 3 displays, for each participant, results of gait analysis during level locomotion over periods of 20 steps or more. Minimum, maximum, mean, and standard deviation of the duration of locomotion period are reported, as well as the number of bouts and analyzed gait cycles. The following gait parameters are shown as mean ± standard deviation (SD): stride velocity, stride length, maximum heel clearance, minimum toe clearance, and gait cycle time variability. The total number of turning steps is also featured in this table.

Table 4 shows the number of stairs and incline walking bouts (non-level locomotion), along with the total duration and number of steps taken during these walking activities.

**Figure 6:**
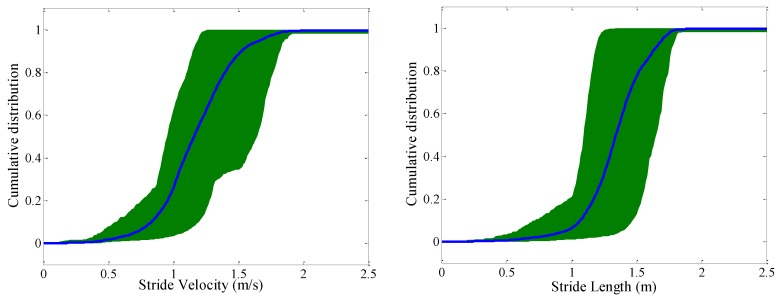
**Left**: stride velocity distribution; **Right**: stride length distribution as mean (thick line) and 5th/95th percentile shading across all subjects.

To illustrate the range in walking performance, gait speed (stride velocity) and stride length profiles are shown in Figure 6. The cumulative distributions were obtained from the cumulated sum of the probability distributions of each subject. Subsequently, the average cumulative distribution (thick line) was calculated as the average of the cumulative distributions from each subject, and the shading represents the area between the 5th and 95th percentiles of the cumulative distributions.

**Figure 7:**
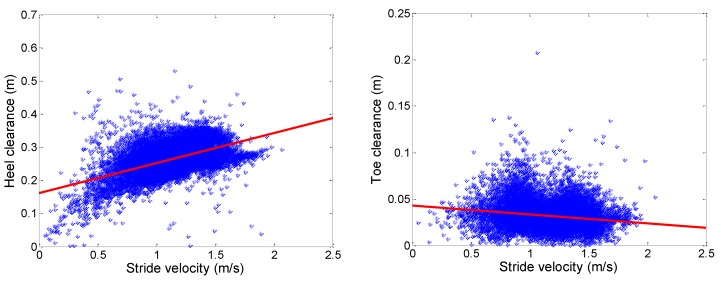
Maximum HC (**left**) and minimum TC (**right**) as a function of stride velocity for all analyzed steps.

Foot clearance is a novel parameter measured in daily life in this study. To highlight the importance of measuring this parameter, Figure 7 shows the relationship between stride velocity and maximum HC /minimum TC, respectively. Pearson’s correlation coefficients reveal moderate positive correlation between HC and gait speed (r = 0.50; *p* < 0.001) and weak negative correlation between minimum TC and gait speed (r = −0.18; *p* < 0.001).

### 3.3. Activity Barcodes, Complexity Metric and Activity Distribution

Table 5 presents individual barcodes constructed for each participant and the corresponding Lempel-Ziv complexity obtained from the instrumented shoes and the reference system, respectively. The correlation between the reference and the instrumented shoes barcodes is considered as strong (r = 0.76, *p* < 0.05).

The correlation between complexity evaluated by instrumented shoes and relevant gait parameters was calculated to shed light on the complementarity of behavioral complexity and gait analysis. The Lempel-Ziv complexity showed little to no correlation with mean stride velocity (r = 0.02, *p* = 0.96), stride length (r = −0.12, *p* = 0.75), max HC (r = −0.05, *p* = 0.88) and min TC (r = −0.28, *p* = 0.43). However, this metric was strongly correlated to the number of gait bouts with more than 20 cycles (r = 0.91, *p* < 0.001) but not with the mean duration of these gait bouts (r = −0.24, *p* = 0.50) nor their maximum duration (r = 0.14, *p* = 0.71).

### 3.4. Evaluation of System Comfort

A total of nine scores from the 10 participants were collected. Missing data is due to the fact that assessment of comfort was introduced to the study protocol only after the first data collection. The scores are distributed as follows: 10, 9, 10, 10, 10, 8, 10, 10, 9, indicating good overall satisfaction (mean 9.6 ± 0.7).

## 4. Discussion

This study presents evidence supporting the feasibility and validity of using an instrumented shoes system to monitor and classify activity during daily life in community-dwelling elderly subjects. Two algorithms were combined in order to provide both a coarse grained activity classification and fine-grained gait analysis towards a comprehensive evaluation of real-life physical behavior. Besides results of the system’s validation, several metrics were proposed to characterize various aspects of daily life physical behavior. Those included postural allocations, locomotion bouts distribution, gait features such as foot clearance and stride velocity, as well as complexity of physical behavior. These aspects are innovative since their previous application has been limited to the laboratory environment. The use of instrumented shoes for activity classification and gait analysis has not yet been demonstrated in daily life, especially the validation of the activity classifier in real life conditions without the presence of an observer. Therefore this study extends previous findings from structured protocols to the real world.

### 4.1. Activity Classification

The main validation outcome of this study pertains to the activity classification algorithm that performed with accuracy as high as 93% in real-life condition, a performance similar to the reference system used for its validation. This result compares favorably to those reported in previous studies on validation of activity classification in a real-life setting. Indeed, studies that used sensors on multiple body locations reported global accuracies ranging between 84% and 89% [14,17,18], whereas studies using single sensor systems reported accuracy from 76% to 80% [10,16,22]. These comparisons further emphasize the advantage of using combined inertial and pressure sensing at the foot level as a single location solution. In this study under real-world conditions, global accuracy of 93% was slightly lower than the 97% obtained with the semi-structured protocol validation [30]. This difference is negligible and appears congruent with similar worsened performance observed in previous studies when classification algorithms validated in lab or semi-structured conditions were applied to data collected under real-life conditions [18,22,43]. This disparity can be explained by the more limited range in both the type and intensity of structured activity assessed during these protocols, as shown in a previous study [44].

Lowest performances were observed for *standing* (88% sensitivity) periods. This slightly low sensitivity ensued mainly from misclassifications of *standing* as *sitting/lying*. Indeed, a couple of transitions from *sitting* to *standing* were not correctly detected and resulted in two relatively long periods of *sitting* classified as *standing*. A dedicated postural transition analysis can provide reliable information on the origin of such misclassifications. Furthermore, misclassifying *standing* into *walking* and vice-versa occurred for shortest locomotion periods (~3–5 steps), as well as from a small systematic difference between the two systems in defining start/end of locomotion periods. However, longer (i.e., 20 steps or more) locomotion periods were almost equally identified by both systems. The sensitivity and precision of *walking* were higher than 90%, reaching similar performance compared to that of the reference system [25]. In terms of locomotion, it would be interesting to study the effect of sensor calibration on the TO detection, as well as evaluating this detection using a calibration free method [45] or by combining inertial data with force data from the insole at each candidate gait cycle to confirm its occurrence.

*Sitting* and *lying* activities were combined into a single activity type. This limitation of the system is arguably relative since *lying* is an activity class that will rarely be observed by the system. In fact, people in their home environment would frequently remove their shoes before going to bed. A further relative limitation relates to the assumption we made that energy expenditure of *lying* and *sitting* are similar [46]. It could be hypothesized that during *lying*, the insole should measure negligible force under the feet, and this in turn could be used to classify *lying*. However this remains to be investigated.

### 4.2. Gait Analysis

Instrumented shoes have been used in the past for gait analysis of level walking during locomotion tests in clinical or laboratory environment [27,34,47,48]. This is, to the best of our knowledge, the first study combining activity monitoring and gait analysis using a single instrumented shoes system in daily life. Considering the high accuracy of the activity classification algorithm as well as the possibility to distinguish level walking from stairs or ramps, gait analysis could be performed on correctly classified level walking bouts with a sufficient number of steps.

In terms of spatio-temporal gait analysis, we showed the potential to provide reliable gait parameters for steady-state gait (periods with >20 steps). In this study, the mean stride velocity, stride length, maximum HC and minimum TC were similar to normative values obtained for an age matched cohort of healthy elderly subjects performing a 20 m gait test in laboratory conditions [49]. Stride velocity, stride length and cadence measured during daily activity are significantly and prospectively associated with falls in elderly subjects as shown in a recent study [3]; instrumented shoes providing accurate estimation of these parameters could therefore be further used for fall prediction. Another original contribution of this study is to show the feasibility to record foot clearance parameters in daily life. To the best of our knowledge, these parameters have not yet been retrieved in other than clinical or gait lab settings, and never over extended periods such as performed in the current study. There is a major interest in obtaining clearance data from daily life especially since this parameter expresses the highest variance in gait data obtained from elderly subjects [47]. In the present study, clearance parameters were moderately (HC) and weakly (TC) correlated to stride velocity, a result similar to observations made in laboratory-based gait analysis over 20 m in an age- and health-matched older population [49]. Therefore, these parameters could provide new insights on a subject’s performance in addition to stride velocity; while simultaneously playing a crucial role in obstacle negotiation and fall avoidance. This could be complementary to context aware systems where the presence of obstacles can be detected. Furthermore, the instrumented shoes could be equipped with additional sensors such as infrared or ultrasound, which have shown the possibility to detect obstacles at relatively short distances from the foot [50]. However, the main interest in measuring clearance parameters lies in their longitudinal evolution, where a decrease in these parameters could indicate higher risk of fall.

The cumulative distribution of locomotion periods provides a good illustration of a subject’s overall mobility performance. In our study, this distribution varied substantially from one participant to another (Figure 5). A shift to the left of this sigmoid curve would indicate reduced occurrence of long periods of walking. Around 94% of locomotion periods were under one minute. The results vary somewhat compared to the literature; for example, Brodie et al. reported that almost 90% were less than a minute [31], whereas Orendurff et al. reported 81% of locomotion periods under one minute [38]. This is arguably due to the longer monitoring time in these two studies. However, the cadence distribution in the current study revealed a bimodal pattern that is similar to the result by Brodie et al. [31], even though the cadence peaks differ slightly (again, possibly due to monitoring time). Incidentally, when locomotion periods were separated by number of steps (<20 vs. 20 or more steps), the cadence modes were similar to those reported in [31]. This result in itself is important because it underpins the hypothesis that locomotion strategies are different between short and long bouts of walking. Gini index [51] or Kolmogorov-Smirnov distance [52] between distribution curves could be further used for the comparison of activity behaviors between subjects with different health conditions, as well as comparisons within the same individual over time to identify change in her/his activity level that could flag an underlying health problem.

### 4.3. Physical Behavior Complexity

The high correlation of Lempel-Ziv complexity values obtained from instrumented shoe barcodes with the reference system justifies the use of the instrumented shoes to assess physical behavior complexity. It should be noted that there is a slight discrepancy due to the few errors of activity classification between the two systems, mainly pertaining to misclassifications of *walking* into *standing* and vice versa. A systematic underestimation of the Lempel-Ziv complexity metric by the instrumented shoes was observed. This could be explained by the lower number of states in the instrumented shoes barcodes (maximum of 14) compared to the reference system (maximum of 18). Still, results strongly suggest the potential application of instrumented shoes to assess physical behavior complexity in different populations of older persons. For instance, this system could be used to monitor progresses in patients undergoing rehabilitation. Another potential application could be to evaluate the potential positive or negative effects of a new medication regimen on mobility and activity over daytime periods.

Interestingly, there was no strong association between gait parameters (stride velocity, stride length, heel clearance and toe clearance) and the Lempel-Ziv complexity values. In contrast, this measure of complexity was highly correlated (r = 0.91, *p* < 0.001) with the number of steady-state locomotion bouts (i.e., 20 steps or more). This result strongly suggests the complementarity of activity pattern analysis and classical gait analysis. For example, participant 7 who achieved the highest average stride velocity had 14 steady-state locomotion bouts only whereas participant 8 who had the highest complexity value completed 60 bouts of steady-state locomotion but had a mean stride velocity lower than 1 m/s. Thus, the complexity metric adds information to mobility assessment by quantifying physical behavior that cannot be achieved by looking at activity distribution/step counts or spatio-temporal gait parameters.

### 4.4. System Evaluation and Drawbacks

Participants gave highly positive feedback on the usability of the instrumented shoes in terms of comfort. Although the methodology used is subject to limitation (participants providing socially desirable answers, assessment not based on an exhaustive, previously validated questionnaire), these results can be considered as preliminary positive and encouraging from end-users of the instrumented-shoes system. Additional investigation of other dimensions such as its easiness of use or end-users’ concern about robustness or reliability need to be considered in the future.

Some additional limitations of our study should be noted. The number of participants is limited and the recording time only covers 4 h. All participants were fit and living independently, therefore results of this study do not reflect physical behavior and gait performance in frailer older persons who are the ultimate target population of this system. However, results of this feasibility study are sufficiently encouraging to further consider additional investigations such as including more participants from other populations (e.g., frail elderly or stroke patients), as well as performing longitudinal studies within the same individuals (e.g., monitoring of activity at baseline and at the end of rehabilitation). In our previous study it was also shown that stairs, ramps, and elevators can be recognized [33]. The validation of these events was not possible in the present study because the reference system used was minimized to lessen intrusiveness and therefore did not include an event marker to provide information on these activities as was the case in our previous validation study [33]. However, since the detection of elevation change depends mainly on barometric pressure variations, it would be possible to add the detection of such events in real life without compromising the accuracy of the classifier. These activities can be added to the activity barcode to enrich the complexity metric. In fact, non-level locomotion has different energy expenditure requirements compared to level walking and it would be extremely interesting to further compare barcodes in persons who frequently engage in such activities to those who rarely do. The calibration of the insoles relied on a simple technique that required 5 s of quiet standing; this was useful for the estimation of total force used in the algorithm. For accurate force measurements, it would be interesting to calibrate each insole sensor individually in static and dynamic conditions.

## 5. Conclusions

We have presented and validated an instrumented shoe system for activity and gait monitoring of older adults in daily life. The activity classification algorithm proved to be highly accurate in identifying basic activities (*siting/lying,* *standing*, and *walking*) and in distinguishing different types of locomotion (*incline walking* and *stairs climbing*). The feasibility of classifying daily life activity in elderly subjects was demonstrated and the system was capable of evaluating locomotion by performing highly detailed gait analysis on locomotion periods of sufficient durations. An additional important contribution of this study is to show that clinically relevant gait parameters such as stride velocity, stride length, cadence and their distribution during the period of recording can be extracted from instrumented shoes data. Moreover, some original gait parameters, such as foot clearance, were detected for the first time in daily life situation. The outcome measures from the instrumented shoes can also be accurately combined in an activity barcode embedding the complexity of daily life activity. This information on complexity appears to extend and enrich the type of information on physical behavior beyond what is usually assessed. The instrumented shoes were judged comfortable to use and did not hinder the movement of participants during daily life. Overall, these results are promising to contemplate further applications of this system in more frail and diverse populations.

## Figures and Tables

**Table 1:** Coding activities based on duration and intensity thresholds; d: duration, cad: cadence.

Activity Type	Activity Duration	Activity Intensity	Numeric Code
Sitting/Lying	-	-	1
Standing	-	-	2
Walking	d < 30 s	cad < 50	3
		50 < cad < 80	4
		80 < cad < 140	5
		140 < cad	6
	30 < d < 120 s	cad < 50	7
		50 < cad < 80	8
		80 < cad < 140	9
		140 < cad	10
	120 < d	cad < 50	11
		50 < cad < 80	12
		80 < cad < 140	13
		140 < cad	14

**Table 2:** Confusion matrix and classifier performance compared to reference activity. Each unit represents a 6 s activity epoch.

	Predicted	Sitting/Lying	Standing	Walking
Reference				
Sitting/Lying		9789	87	11
Standing		566	6788	402
Walking		1	420	3986
Sensitivity		0.99	0.88	0.90
Specificity		0.99	0.93	0.98
Precision		0.95	0.93	0.91
F-score		0.97	0.90	0.91

**Table 3:** Gait characterization from level walking periods of at least 20 steps. Reported values are mean ± SD unless otherwise stated.

Participant	Duration (s) (min/max)	Duration (s)	# Bouts	# Gait Cycles	Stride Velocity (m/s)	Stride Length (m)	Heel Clearance (m)	Toe Clearance (m)	Variability (%)	# Turning Steps
1	13.86/190.48	56.73 ± 49.34	34	1419	1.07 ± 0.19	1.33 ± 0.15	0.28 ± 0.04	0.02 ± 0.01	8.19 ± 7.66	232
2	12.82/431.82	48.65 ± 73.74	34	1346	1.29 ± 0.20	1.43 ± 0.14	0.30 ± 0.04	0.03 ± 0.01	8.83 ± 11.05	240
3	14.59/284.61	94.80 ± 75.01	18	1284	0.97 ± 0.16	1.22 ± 0.12	0.27 ± 0.03	0.04 ± 0.02	6.56 ± 3.54	102
4	15.04/295.95	58.99 ± 63.54	50	2213	1.12 ± 0.20	1.32 ± 0.12	0.27 ± 0.02	0.03 ± 0.01	7.21 ± 6.11	390
5	12.97/60.54	24.36 ± 10.69	31	538	1.07 ± 0.34	1.22 ± 0.32	0.26 ± 0.05	0.04 ± 0.01	11.21 ± 10.12	176
6	10.68/130.64	35.07 ± 27.53	39	1082	1.28 ± 0.25	1.37 ± 0.22	0.25 ± 0.03	0.03 ± 0.01	9.69 ± 11.73	283
7	12.63/162.40	29.04 ± 38.67	14	307	1.47 ± 0.38	1.55 ± 0.26	0.27 ± 0.03	0.03 ± 0.01	9.51 ± 7.97	96
8	13.16/275.15	53.49 ± 64.30	60	2708	0.99 ± 0.16	1.07 ± 0.12	0.22 ± 0.02	0.03 ± 0.01	7.03 ± 6.30	345
9	12.97/368.34	49.77 ± 55.64	50	1939	1.37 ± 0.18	1.60 ± 0.16	0.31 ± 0.03	0.03 ± 0.01	7.85 ± 8.11	392
10	15.22/277.31	84.35 ± 96.03	11	735	1.06 ± 0.12	1.26 ± 0.10	0.22 ± 0.01	0.04±0.01	8.79 ± 9.04	65

**Table 4:** Non-level locomotion periods. TD: total duration in seconds.

Participant	Upstairs			Downstairs			Uphill			Downhill		
	Bouts	TD (s)	Steps	Bouts	TD (s)	Steps	Bouts	TD (s)	Steps	Bouts	TD (s)	Steps
1	3	47.27	43	7	149.54	117	2	99.49	71	1	45.21	36
2	2	52.60	48	5	393.09	374	1	35.49	33	1	32.85	31
3	0	0	0	3	65.95	55	1	36.83	27	0	0	0
4	3	95.45	84	5	243.36	205	1	67.18	55	0	0	0
5	2	55.73	48	3	60.60	53	0	0	0	0	0	0
6	7	272.51	253	5	181.08	177	0	0	0	0	0	0
7	6	40.49	33	2	29.91	26	0	0	0	0	0	0
8	0	0	0	0	0	0	0	0	0	0	0	0
9	11	193.77	162	14	168.33	150	2	16.39	16	0	0	0
10	3	96.05	76	1	21.08	17	0	0	0	0	0	0

**Table 5:** Subject specific activity barcodes. The scale on the right indicates the activity intensity, starting from 1: sitting, 2: standing, 3–14: walking with different cadences and locomotion period durations. Lempel-Ziv complexity values for each subject and for each activity monitoring system are shown.

	Lempel-Ziv Complexity		Activity Barcodes from Instrumented Shoes
	Instrumented Shoes	Reference	Scale: 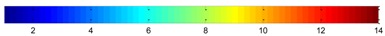
P1	0.286	0.480	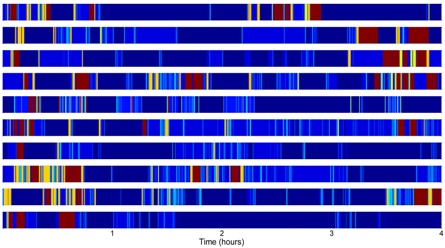
P2	0.3	0.521	
P3	0.294	0.449	
P4	0.383	0.628	
P5	0.305	0.588	
P6	0.367	0.575	
P7	0.289	0.526	
P8	0.409	0.566	
P9	0.371	0.579	
P10	0.258	0.339	

